# Are more observational studies being included in Cochrane Reviews?

**DOI:** 10.1186/1756-0500-5-570

**Published:** 2012-10-16

**Authors:** Hans Christian Kongsted, Merete Konnerup

**Affiliations:** 1 Department of Economics, University of Copenhagen, Øster Farimagsgade 5, DK-1353, Copenhagen K, Denmark; 2 The Campbell Collaboration’s steering group and Trygfonden, Lyngby Hovedgade 4, 2. sal, DK-2800, Kongens Lyngby, Denmark

**Keywords:** Systematic reviews, Observational study, Randomized controlled trial

## Abstract

**Background:**

Increasing the scope of an evidence based approach to areas outside healthcare has renewed the importance of a long-standing discussion on randomised versus observational study designs in evaluating the effectiveness of interventions. We investigate statistically if an increasing recognition of the role of certain nonrandomised studies to support or generalize the results of randomised controlled trials has had an impact on the actual inclusion criteria applied in Cochrane reviews.

**Methods:**

We conduct an on-line search of the Cochrane Database of Systematic Reviews (CDSR) and divide all Cochrane reviews according to their design inclusion criterion: (A) RCTs only or (B) RCTs and (some subset of) observational studies. We test statistically whether a shift in the proportion of category B reviews has occurred by comparing reviews published before 2008 with reviews published during 2008/09.

**Results:**

We find that the proportion of Cochrane reviews choosing a broader inclusion criterion has increased, although by less than two percentage points. The shift is not statistically significant (P = 0.08).

**Conclusions:**

There is currently not sufficient data to support a hypothesis of a significant shift in favour of including observational studies, neither at the aggregate level nor at the level of individual Review Groups within the Cochrane Collaboration.

## Background

“*Evidence based medicine is the conscientious, explicit, and judicious use of current best evidence in making decisions about the care of individual patients*” [1]. This is the oft-cited definition of evidence based medicine (EBM) [2]. As one of the most visible parts of the EBM movement, the Cochrane Collaboration [3] was established in 1993 as an international not-for-profit independent organization. It has produced around 4,000 systematic reviews of the effects of healthcare interventions. More recently, there have been efforts to extend the evidence based approach to policy areas outside healthcare. The Campbell Collaboration [4,5] was set up in 2000 to facilitate systematic reviews of the effects of social interventions in areas such as social work, education and crime & justice. (Its close cooperation with the Cochrane Collaboration is witnessed by the recent October 2010 joint colloquium [6]).

This expansion of scope of the evidence-based approach has renewed the long-standing discussion of the role of randomised versus observational study designs [7-27]. A key feature of EBM is advocating a hierarchy of what constitutes “*current best evidence*” on effectiveness. Evidence from systematic reviews of several high-quality randomised controlled trials (RCTs) rank at the top, followed by single RCTs, and then observational studies [28]. Randomised studies are thus widely regarded as *a priori* most likely to produce an unbiased estimate of effect [24]. There is, however, an increasing recognition of the role of nonrandomised studies, either to support the results of RCTs, or in cases where there are no existing RCTs [24]. This role is likely to be much emphasized when assessing the effectiveness of non-healthcare public policies due to the relative shortage of RCTs in such areas.

Despite this focus on primary study design, there appears to be little if any quantitative information on how the actual inclusion criteria applied in systematic reviews of healthcare interventions have evolved over time. By producing such evidence based on the existing reviews of the Cochrane Database of Systematic Reviews (CDSR), our primary aim is to inform the discussion of the roles of randomised and nonrandomised studies for an evidence-based approach in fields outside healthcare. Due to similarity of the setups of the Cochrane and Campbell Collaborations [5], we argue that analysing the former is informative for the discussion on how to implement systematic reviewing of social interventions within the latter. It is known that the Cochrane Collaboration has a stronger focus on RCTs when compared to systematic reviews produced by others in the medical literature [29,30]. Still, the analysis of the CDSR is arguably of interest in itself. It has risen to the top-10 in the Thomson ISI “MEDICINE, GENERAL & INTERNAL” category [3] in terms of its impact factor and it is thus an increasingly influential source of information within the medical literature.

Specifically, we examine how the criteria for inclusion of different primary study designs have varied both over time and across different medical fields. The fundamental distinction that we focus on is between experimental and observational designs [24]. As most of the existing literature, we will use the terms observational and non-randomised interchangeably.

The production of Cochrane reviews and the subsequent editorial review process is guided (but not ruled) by the Cochrane Handbook [31]. The overarching principle of the current Handbook (section 5.5) is that “*[r]andomisation is the only way to prevent systematic differences between baseline characteristics of participants in different intervention groups in terms of both known and unknown (or unmeasured) confounders…”*[31]*.* Still, in accordance with this principle, the Handbook (section 13.1.2) allows the inclusion of non-randomised studies “*a) [t]o examine the case for undertaking a randomised trial by providing an explicit evaluation of the weaknesses of available [non-randomised studies] … b) [t]o provide evidence of the effects (benefit or harm) of interventions that cannot be randomised, or which are extremely unlikely to be studied in randomised trials … c)[or to] provide evidence of effects (benefit or harm) that cannot be adequately studied in randomised trials, such as long-term and rare outcomes, or outcomes that were not known to be important when existing, major randomised trials were conducted*” [31].

In analyzing if there are changes in the actual inclusion criteria applied in Cochrane reviews, we pay special attention to the time of publication of version 5 of the Cochrane Handbook [31] in September 2007. It devotes a full chapter to the role and quality of observational studies. Previous versions of the Handbook (up to and including version 4.2.6) relegated the discussion to a short appendix. While we focus on end-2007, the exact cut-off point in time should not be seen as a main issue. We conduct sensitivity analyses for alternative dates of a potential shift and investigate more generally if there was a shift in research design inclusion criteria of Cochrane reviews published towards the end of the recent decade.

The above list of cases in which non-randomised study designs can be included, could apply more often in some medical subfields than in others. We therefore also investigate the set of reviews that originates from each of the so-called Cochrane Review Groups (CRGs) set up in the Cochrane Collaboration to manage the editorial process within a specific medical subfield.

## Methods

### Data sources and definitions

We conducted an on-line search of the Cochrane Database of Systematic Reviews (CDSR), issue 3, 2009. We split the total pool of Cochrane reviews into two mutually exclusive and exhaustive categories according to their choice of which research designs to include: (A) RCTs only or (B) RCTs and (some subset of) observational studies. We identified category (B) reviews among those generated by the search string “non NEXT random* OR nonrandom* OR non NEXT rct” applied to the full text of the review. The Cochrane Handbook uses the term “non-random” when discussing observational studies. Our search string thus identifies the reviews that explicitly consider whether or not to include such studies.

The identified reviews constitute a *gross* measure of category (B) reviews in the sense that some reviews simply mention observational studies but decide not to include them. We therefore manually analyzed each review within the gross selection and identified those reviews that actually allowed for the inclusion of (some subset of) observational studies. The manual analysis was based on the subsection “*Types of Studies*” in the Methods section of the standardized lay-out for Cochrane reviews. We checked inter-coder reliability by having the two authors independently assign categories to 1 review randomly selected in every 25 reviews within the gross selection. There was agreement on the proper categorization of all randomly sampled reviews. As a further check for possible misclassification, we also applied our manual search to the first 25 reviews listed when we simply sorted all Cochrane reviews alphabetically by title. Eight of these were already in our selection. One had been withdrawn. Of the remaining 16 reviews, all included only RCTs or quasi-RCTs, none allowed for the inclusion of observational studies. This validates our gross selection criteria.

### Statistical analysis

We analyze the full period of the CDSR as well as two sub-periods – before or after January 1^st^, 2008 – as distinguished by the year of publication of the review. The editorial process of reviews published before January 1^st^, 2008, was fully or predominantly guided by the Handbook version 4.2.6 (or earlier versions). For reviews published after January 1^st^, 2008, version 5 of the Handbook [31] from September 2007 has definitely been available during long stretches of the review production process. We use likelihood ratio *χ*^2^ tests throughout. A P-value of less than 0.05 was considered to indicate statistical significance.

### Sensitivity analyses

We subdivided category (A) into an (A1) category of reviews that use a strict RCT-only inclusion criterion and an (A2) category of reviews that additionally allow for the inclusion of quasi-randomised trials. The Cochrane Handbook [31] (section 13.1.1) uses the term quasi-randomised trial to signify “*inappropriate randomisation strategies*” (e.g. alternate or case file number) where bias is potentially introduced through unrecognized correlation between the sequence generation mechanism and the outcome(s) of interest. While quasi-randomised studies cannot be considered as a proxy for observational studies, they are a “grey zone” in terms of proper randomisation. For the main analysis, we grouped categories (A1) and (A2) together. We perform a sensitivity analysis by eliminating category (A2) from the analysis altogether.

In order to increase the power of our analysis against changes that took place gradually, we also present a sensitivity analysis that compares 2008/09 reviews to the reviews that were published prior to 2006.

## Results and discussion

Table 1 illustrates how the total pool of Cochrane reviews is distributed according to study design inclusion criteria. The reviews are divided into three categories: (A1) Reviews that only allow for RCTs; (A2) for RCTs and quasi-randomised designs; or (B) for RCTs, quasi-randomised designs and (some subset of) observational study designs. The table also provides separate distributions for reviews published prior to 2008 or during 2008/09.

**Table 1:** Distribution of Cochrane reviews over inclusion criteria

**Study design inclusion criteria**	**Total**	**of which published:**	
		**pre-2008**	**2008-2009**
	**Number (per cent)**	**Number (per cent)**	**Number (per cent)**
(A1) Randomised controlled trials	3,398 (87.2)	2,563 (87.9)	835 (85.2)
(A2) Randomised controlled trials and quasi-randomised trials	277 (7.1)	199 (6.8)	78 (8.0)
(B) Randomised controlled trials, quasi-randomised trials, and (some subset of) observational studies	222 (5.7)	155 (5.3)	67 (6.8)
Total	3,897 (100.0)	2,917 (100.0)	980 (100.0)

Source: Cochrane Database of Systematic Reviews, Issue 3, 2009. A list of the categories assigned to individual reviews is found in the Additional file 1.

The vast majority of existing Cochrane reviews – 87 per cent – belong to category (A1). They only allow for the inclusion of RCTs. Category (A2) reviews which in addition include quasi-randomised designs, add another seven percentage points. Approximately six per cent of all Cochrane reviews are category (B) reviews that allow for the inclusion of (some subset of) observational study designs.

**Figure 1:**
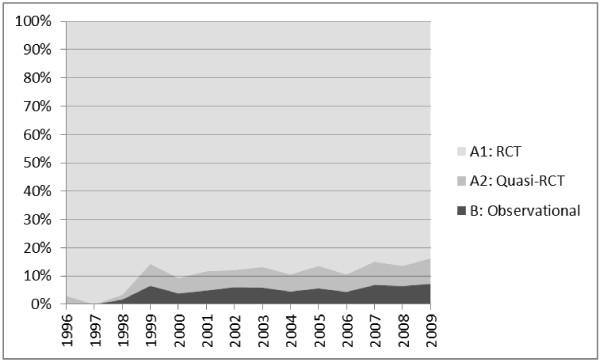
Distribution of Cochrane reviews across inclusion criteria.

Figure 1 illustrates the evolution of this distribution since the mid 1990ies. An eye-ball inspection seems to suggest that the proportion of category (B) reviews is trending upwards although quite slowly. The early part of the figure is based on very few reviews – hence the large variance.

Comparing the reviews published pre-2008 to reviews published in 2008/09 (Table 1), we find that the category (B) proportion has increased by less than two percentage points. The change is not statistically significant (P = 0.08). Likewise, a statistically insignificant result (P = 0.07) is obtained when we eliminate “grey zone” category (A2) reviews from the analysis. Consequently, there is currently not sufficient data at the aggregate level to statistically reject that the proportion of Cochrane reviews which include (some subset of) observational studies has remained constant.

**Figure 2:**
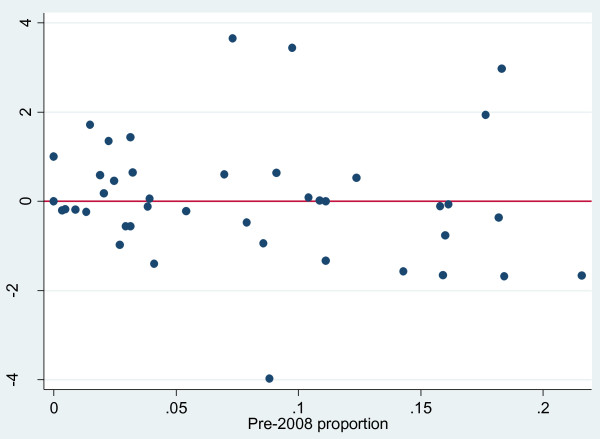
**Production of observational-inclusive (category B) reviews by Review Groups in the Cochrane Collaboration.** Note for Figure 2: Vertical axis: Actual number of category (B) reviews published in 2008/09 minus the number to be expected given pre-2008 proportions. Horizontal axis: Pre-2008 proportion of category (B) reviews. Example: a negative number means that a review group has produced fewer category (B) reviews in 2008 or 2009 than would be expected from its production prior to 2008.

Despite the insignificant finding at the aggregate level, there is still a possibility of diverging trends within different medical subfields. To investigate this, Figure 2 orders the CRGs horizontally by increasing pre-2008 proportion of category (B) reviews. (A similar picture emerges when eliminating the “grey zone” category (A2) reviews.) Individual subfields are seen to range widely from a low of zero category (B) reviews in the “Haematological Cancer Group” and the “Hypertension Group” to a high of 22 per cent in the “Gynaecological Cancer Group”. The variation across fields confirms the hypothesis that the supplementary role of observational studies differs greatly between fields.

Figure 2 makes it possible to do an eye-ball inspection of the possible presence of a structural shift at the level of each individual Cochrane Review Group. It shows the difference between the *actual* number of category (B) reviews published in 2008/09, and the number to be *expected* had the pre-2008 proportions remained unchanged. Values larger (smaller) than zero indicate that the proportion of category (B) reviews in a CRG increased (decreased).

The differences between actual and expected changes are seen to be fairly equally divided between increases (21 CRGs) and decreases (28 CRGs). (The “Childhood Cancer Group” had no pre-2008 reviews and is therefore not included.) The graphical interpretation of Figure 2 needs to take account of the fact that the category (B) proportion has limited “downside” within previously low-proportion CRGs. Among the ten CRGs having the highest pre-2008 proportion of category (B) reviews, eight show a declining proportion (Figure 2). Increases are found only in two of the top-ten CRGs: The “Effective Practice and Organisation Group” and the “Injuries Group”. Both groups focus on behavior modifying interventions rather than RCT-intense pharmacological interventions.

Formal testing of significance within subfields requires a sufficiently large number of reviews to be expected in 2008/09 (given unchanged pre-2008 proportions). This can be achieved by a large review production in general, a high pre-2008 proportion of category (B) reviews, or both. None of the individual CRGs currently qualify for testing under a standard requirement of at least five expected observations.

The strict comparison of pre- and post-2008 reviews could potentially miss a shift that happened gradually. First, a number of reviews published in 2008/09 had their protocol formally approved under the guidance of Handbook version 4.2.6. The protocol includes the very study design criterion on which we base our categorization of reviews. Cochrane reviews are approved sequentially (title, protocol, review) and typically over several years. Statistically correcting for this potential source of bias must await the publication of more reviews governed by Handbook version 5 “from start to finish”. Secondly, any change within an individual CRG could have predated the publication of Cochrane Handbook version 5. Given that the Handbook is a guide, not a rule book, a change in the rate of inclusion of observational studies could have been present in actual review practice already during years prior to the actual publication date of Handbook version 5.

As a sensitivity analysis, we therefore introduced a two-year separation between the sub periods, comparing 980 2008/09 reviews and 1,755 reviews published prior to 2006. A very similar picture emerged from this analysis: The aggregate proportion of category (B) reviews does increase but still not significantly; 20 CRGs show increasing category (B) proportions, 29 show decreases; and there is not enough data to allow formal significance testing at the level of the individual CRGs.

## Conclusions

The methodological debate on the role of observational studies in formalized research synthesis has been ongoing in medical research. The present analysis documents how the formal guidelines of the Cochrane Collaboration have impacted on actual review practice. The main conclusions are threefold.

First, the overall distribution of Cochrane reviews according to inclusion criteria reflects a clear and dominant preference for RCTs. Second, the analysis showed a large range of variation in the rate at which reviews allow for the inclusion of observational studies across different medical subfields. Third, we found no significant evidence of any subsequent shift in actual review practice. This conclusion holds irrespective of whether we use the time of publication of the Cochrane Handbook version 5 to date the potential shift; allow for a more gradual change by separating the sub periods by a window of two years; or exclude reviews that allow for quasi-randomised trials but not observational studies from the analysis.

These conclusions have implications for extending a similar “industrial scale” [32] review production initiative to public policy areas outside healthcare. Even within the realm of healthcare interventions, there is no “one size fits all” prescription. The need for supplementing the evidence from randomised controlled trials with supporting evidence from observational studies varies greatly across medical subfields. Most likely, this will apply to an even greater extent for interventions outside healthcare. Moreover, the fact that we found no significant trends either at the aggregate level or within specific medical subfields, suggests that the rate of inclusion of observational studies is dictated by field-specific needs which remain fairly constant over time, rather than the ups and downs of ongoing methodological discussions.

## Competing interests

The opinions expressed in this paper do not necessarily reflect the views neither of the Campbell Collaboration as such nor of other members of the Campbell Collaboration’s steering group. Merete Konnerup has been reimbursed for attending the Joint Cochrane-Campbell Colloquium by the Campbell Collaboration. Hans Christian Kongsted has nothing to declare.

## Authors’ contributions

The authors have jointly collected the data and drafted the text. HCK performed the statistical analyses. Both authors read and approved the final manuscript.

## Supplementary Material

Additional file 1 List of categories assigned to individual reviews. Click here for file
